# Targeted therapy in gastric cancer

**DOI:** 10.1007/s10353-016-0389-1

**Published:** 2016-03-07

**Authors:** G. Jomrich, S. F. Schoppmann

**Affiliations:** Department of Surgery, Gastroesophageal Tumor Unit, Comprehensive Cancer Center (CCC), Medical University of Vienna, Spitalgasse 23, 1090 Vienna, Austria

**Keywords:** Gastric cancer, HER2, Angiogenesis, Targeted therapy, Trastuzumab, Ramucirumab, Chemo-resistance

## Abstract

**Background:**

Gastric cancer is the fourth most common cancer worldwide. Surgery in combination with multimodal therapy provides the only curative therapy until now. The importance of targeted therapy became clear over the last few years. Due to the implication of HER2 and angiogenesis-directed targeted therapies major advances in the treatment of gastric cancer could be reached. Nevertheless, benefits in survival remain unsatisfactory and the development of resistance to monoclonal antibodies is arising.

**Methods:**

A comprehensive and comparative literature research was performed to evaluate the status of HER2 and angiogenesis-directed targeted therapy in gastric cancer.

**Results:**

Up to now, trastuzumab and ramucirumab are the only agents showing remarkable benefits in the therapy for the patients suffering from gastric cancer. The limitations of targeted therapies in gastric cancer are mainly associated with the development of secondary resistance.

**Conclusion:**

Addition of targeted therapy in second-line treatment is beneficial when compared with chemotherapy alone. Nevertheless, results in first-line treatment remain modest. Therefore, new therapeutic agents and combinations in the first-line treatment of gastric cancer are urgently needed and remain to be validated in clinical trials.

## Introduction

Gastric cancer is one of the most commonly diagnosed cancers worldwide. Around 700,000 people die due to gastric cancer every year. This makes gastric cancer the second most common cause of cancer related death in the world [1]. Western countries report a lower incidence of gastric cancer, patients, however, have a higher mortality due to advanced stages of disease at the time of diagnosis [2].

Until now, the only curative therapy option is a multimodal approach in resectable gastric cancer [3, 4]. In metastatic disease 5-year survival rate is very poor with a median overall survival (OS)below 12 month [5].

The human epidermal growth factor receptor 2 (HER2) is one of the most important targets for targeted, anti-cancer therapies. Overexpression and amplification of HER2 in gastric cancer leads to a poor prognosis, due to the initiation of progression and metastasis [1, 6, 7]. A significant benefit in advanced gastric cancer could be observed, using the monoclonal antibody trastuzumab, in combination with chemotherapy [8].

Due to angiogenesis, networks are built up, supplying the tumor with nutrients and oxygen regulated via activator and inhibitor molecules [9–11]. In angiogenesis, the vascular endothelial growth factor (VEGF) family and its receptors (VEGFRs) are of substantial importance. In mammals, the VEGF family members (ligands) are VEGF-A, VEGF-B, VEGF-C, VEGF-D, and placenta growth factor (PGF). The most important signal transducer is VEGFR-2. Through a number of different pathways, VEGFR-2 regulates cell proliferation.

The increasing attention in tumor vascularization leads to the development of antiangiogenic therapies. As a result, various humanized antibodies, such as bevacizumab and tyrosine kinase inhibitors (TKIs), have been approved for anti-cancer therapies. This article describes the actual state of the art therapeutic options in gastric cancer. Further, the potential role of new agents in targeted therapy of gastric cancer will be evaluated and discussed.

## Material and methods

An analytical and comparative PubMed research for targeted therapy in gastric cancer was executed. Results were implemented into a critical analysis and discussion of the state of the art-targeted therapy of gastric cancer.

## Results

### Resectable gastric cancer

Up to date, a multimodal approach, consisting of a combination of surgical resection plus/minus chemo (radio) therapy remains the only potentially curative option in resectable gastric cancer. Chemo (radio) therapy combined with the classical “D2 resection” represents a standard procedure in localized gastric cancer. Extending lymphadenectomy with paraaortic nodal dissection brought no significant benefits concerning survival and/or recurrence [4, 12, 13].

### Adjuvant chemotherapy and chemoradiatio

Before the Japanese ACTS-GC trial was investigated, in more than 30 randomized trials, comparing adjuvant systemic chemotherapy versus surgery alone in resectable cancer, no benefit in patients’ survival could be observed [14–16]. Only the ACTS-GC trial with 1059 patients with stage II or III gastric cancer (oral S-1 versus D2 gastrectomy alone) demonstrated significant improvement in OS [17]. Due to this results could not be found in non-Japanese patients, S-1 is approved in Japan for adjuvant therapy in gastric cancer and in Europe for the treatment of advanced gastric cancer. The results of the Intergroup-0116 trial established adjuvant chemoradiotherapy as a standard treatment for patients suffering from resectable gastric cancer. Due to the Intergroup-0116 trial was criticized because of inadequate surgical procedures the Korean ARTIST trial, which randomly assigned only patients who underwent D2 lymphadenectomy, reassessed adjuvant radiotherapy in gastric cancer [3, 13]. Unfortunately, no significant benefit in Disease Free Survival (DFS) and OS could be observed for the entire study population [18]. Therefore, further investigations and trials, such as the ARTIST-II trial, the CLASSIC trial or the CRITICS trial are needed to find new, beneficial adjuvant therapeutic regiments ([17, 19, 20] Table 1).Author: Please check the edit made in the sentence "results could not be found in non-Japanese patients" conveys/retains the intended meaning.No changes required.Author: Please provide the expansion of the abbreviation "DFS".Disease Free Survival

**Table 1:** Adjuvant chemotherapy for resectable gastric cancer—A summary of major studies. ([3, 17, 19, 21, 22])

Source, year	Adjuvant Chemotherapy	UICC Stage	5-yrs OS (95 % CI) treated	5-yrs OS (95 % CI) control	HR for OS (95 % CI)
Nitti, D., et al, 2006 [22]	Fluorouracil	IB–IV	52 % (42–62%)	51% (41–61%)	0.89 (0.51–1.31)
	Doxorubicin				
	Methotrexat + Leucovorin				
	Fluorouracil	IB–IV	33% (23–42%)	36% (26–45%)	1.05 (0.69–1.41)
	Epirubicin				
	Methotrexat + Leucovorin				
Sasako, M., et al, 2011 [18]	S1	II–III	72 % (68–76%)	61 (57–65%)	0.67 (0.54–0.83)
			**3-yrs OS (95 % CI) treated**	**3-yrs OS (95% CI) control**	**HR for OS (95% CI)**
Macdonald, JS., et al, 2001 [4]	Fluorouracil	n.g.	50% (n.a.)	41% (n.a.)	1.35 (1.09–1.66)
	Leucovorin				
	Radiation				
Sakuramoto, S., et al, 2007 [23]	S1	II–IIIB	80% (76–84%)	70% (66–75%)	0.68 (0.52–0.87)
Bang, YJ., et al, 2012 [20]	Capecitabine	II–IIIB	83% (79–87%)	78% (74–83%)	0.72 (0.52–1.00)
	Oxaliplatin				

*UICC* Union internationale contre le cancer, *yrs* Years, *OS* Overall survival, *CI* Confidence interval, *HR* Hazard ratio, *n.g.* not given

### Neoadjuvant and perioperative chemotherapy

One of the most influential trials investigating perioperative chemotherapy in gastric cancer is the Medical Research Council Adjuvant Gastric Infusional Chemotherapy (MAGIC) trial. The MAGIC trial recruited 503 patients (74 % potentially resectable gastric cancer, 11 % distal esophageal cancer, or 15 % esophago-gastric junction adenocarcinomas) randomized to three cycles of chemotherapy (5-FU, cisplatin and epirubicin) before and after radical resection, compared with treatment with surgery alone. In the chemotherapy arm, a significantly better OS and progression-free survival (PFS) were reported. Beside these results, a higher rate of curative surgery and a reduced tumor size were observed [23]. As a result of the MAGIC trial, perioperative chemotherapy was established as standard regimen in resectable gastric cancer in wide parts of Europe.

### Implication of targeted therapy

Due to improvements in understanding altered molecular events in cancer the discovery of new targets and agents in gastric cancer were possible. Targeted therapy for solid tumors represents a new therapeutic onset. Nevertheless, some notable successes could have been reached. In advanced or metastatic gastric cancer a curative therapeutic onset is exceedingly rare. Still, the focus in advanced stages is on palliation and best supportive care.

### HER2

HER2, a 185-kDa protein, is encoded by a gene located on chromosome 17q21. Overexpression of HER2 in gastric cancer is reported in 6–23 % [7, 24]. Due to carcinogenic processes and adverse pathological features overexpression of HER2 is related to poor prognosis in gastric cancer [25, 26]. Besides its association with clinicopatholgical features, HER2 amplification is a promising target for targeted therapy [27].

**Fig. 1:**
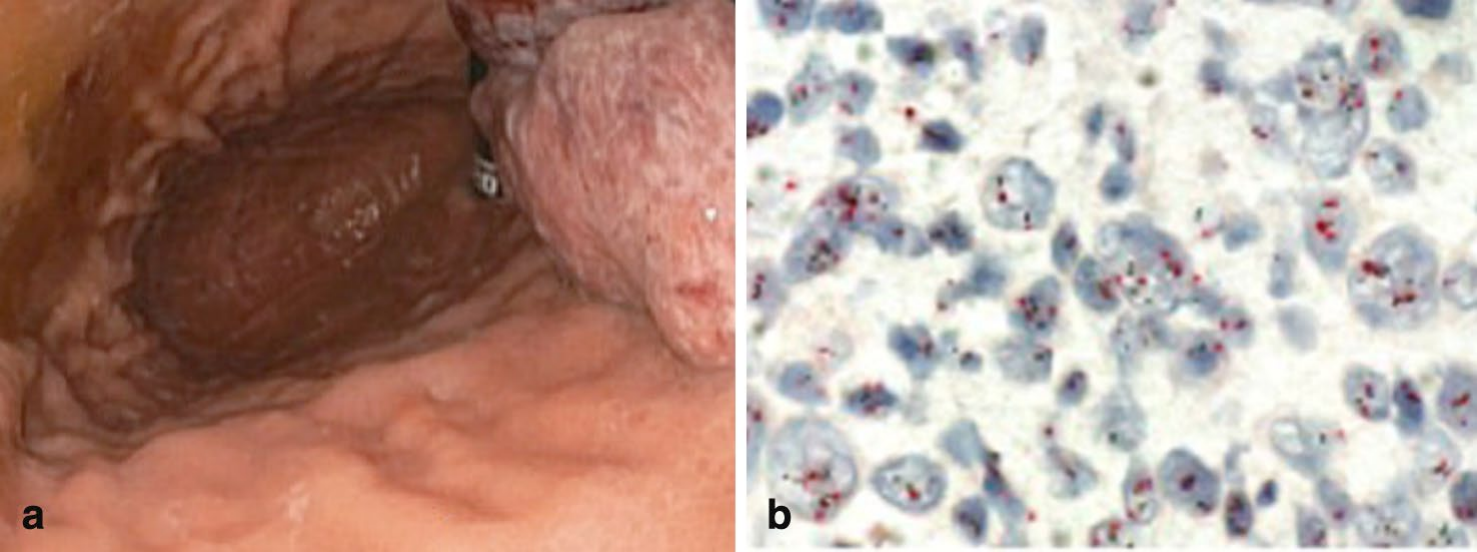
**a** Esophagogastroduodenoscopy showing a gastric adenocarcinoma in the fundus ventriculi. **b** Dual-color in situ hybridization: *red* centromere chromosome 17, *black* the HER-2 gene. Note that the ratio of HER-2 gene copies/centromere 17 is < 2 in the majority of cells. Original magnification x600

In gastric cancer, the expression of HER2 is primarily determined by using immunohistochemistry (IHC) and/or by detecting HER2 gene amplification by in situ hybridization (ISH) as described previously by Hofmann et al. in 2008. Rüschoff et al. reassessed this method in 2010 ([28, 29]; Fig. 1).

### Trastuzumab in gastric cancer

Trastuzumab is the first molecular targeted agent approved as standard therapy in gastric cancer [8, 30]. Trastuzumab induces antibody dependent cellular cytotoxicity. In addition, trastuzumab inhibits HER2 mediated signaling and prevents cleavage of the extracellular domain for HER2. An addition of trastuzumab to conventional cytotoxic chemotherapy in patients with HER2 positive advanced gastric cancer was investigated in the Trastuzumab for Gastric Cancer (ToGA) trial [8]. In terms of tumor response, the ToGA trial showed a clinical benefit in the chemotherapy plus trastuzumab group. Patients receiving chemotherapy and trastuzumab had a significantly better OS. In a reassessment of HER2 expression levels, IHC +++ patients showed the greatest benefit from additional trastuzumab [8]. As a result of this reassessment, the European Medicine Agency (EMA) restricted approval of trastuzumab to patients suffering from IHC +++ or ++/FISH + metastatic gastric or gastro-esophageal junction adenocarcinoma. The National Institute for Clinical Excellence limited its recommendation for trastuzumab to patients showing IHC +++ disease only in the United Kingdom based on this reassessment. Beside these restrictions in Europe, in the United States, the Food and Drug Administration (FDA) has ratified trastuzumab therapy for patients with HER2 overexpression without any further specification.

To overcome these therapeutic insufficiencies, the Japanese multicenter phase II study HERBIS-1 was initiated. HERBIS-1 recruited patients with advanced, HER2-positive gastric cancer. Patients received S1 on day 1–14, cisplatin on day one and trastuzumab on day one of a cycle of 21 days. The response rate, taken from the RECIST trial, was 68 % (95 % confidence interval (CI) 0.54–0.80) and the disease control rate was 94 % (95 % CI 0.84–0.99). The median OS, the PFS and the time to treatment failure (TTF) were estimated at 16.0, 7.8, and 5.7 month, respectively [31].

Upcoming trials to evaluate the value of trastuzumab in gastric cancer are ML25189, a phase II study, planning to recruit 45 patients suffering from resectable and HER2 positive gastric adenocarcinoma or gastro-esophageal junction cancer (type I–III) [32] and the US Radiation Therapy Oncology Group (RTOG)-1010 accrues for a larger, phase III trial with a planned number of 480 patients [33].

### New approaches targeting HER2

The HER2-targeted monoclonal antibody pertuzumab inhibits HER2 heterodimerization. Binding the extracellular domain II of the HER2 receptor, pertuzumab therefore disrupts HER2 dimerization not competing the effect of trastuzumab [34–36].

A phase IIa study of first-line pertuzumab showed only partial response. The combination with trastuzumab, capecitabine, and cisplatin in HER2 positive, advanced gastric cancer patients, brought no remarkable benefits [37].

Lapatinib, a TKI of EGFR and HER2 showed promising results in HER2 positive breast cancer previously. Until today, no significant benefit in gastric cancer could be observed using lapatinib in recently published studies [38].

Trastuzumab emtansine (trastuzumab-DM1), a conjugate of cytotoxic drug maitansine derivate DM1 and trastuzumab, binds microtubules and inhibits their assembly and blocks mitosis, likewise vinca alkaloids do. Using in vitro gastric cancer models, trastuzumab-DM1 shows highly aggressive tumor activity compared with single trastuzumab [39]. Currently, a multicenter phase III study of trastuzumab-DM1 is recruiting patients with HER2-positive advanced gastric cancer after progression after first-line treatment [40].

### Strategies to overcome trastuzumab-resistance

Beside improved outcome in patients with HER2 positive gastric cancer due to trastuzumab, the median duration of response remains moderate. A multitude of patients, suffering from HER2-positive gastric cancer develop secondary resistance to trastuzumab [38]. Thus, a better understanding of the molecular mechanisms in the development of resistance to trastuzumab is urgently needed.

Afatinib is an irreversible inhibitor of EGFR, HER2, and HER4. Afatinib are potentially active against receptors with secondary mutations, resistant to first-generation inhibitors. A phase II study in metastatic HER2-positive trastuzumab refractory esophageal and gastric cancer is underway, currently recruiting patients [41].

The PI3K/Akt/mTOR pathway plays a cruitial role in trastuzumab resistance, dysregulating the HER2 downstream signal [42]. The mTOR inhibitor everolimus inhibits the mTOR/S6K signal, and therefore improves fluorouracil-induced apoptosis in gastric cancer cells with HER2 amplification. A concordant therapy using HER2-targeted agents and everolimus might lead to an improvement in therapy of HER2-positive gastric cancer.

HSP90, an ATP dependent, conserved molecular chaperone, plays a major role in the structural folding and stability of proteins. AUY922, a member of the isoxazole HSP90 inhibitor family, might be a promising agent to overcome secondary trastuzumab resistance [43]. Unfortunately, a clinical phase II study brought no results due to premature termination [44].

### Angiogenesis in gastric cancer

Angiogenesis is a fundamental factor in tumor growth. Due to angiogenesis oxygen and nutrients are supplied to proliferating cancer cells [9]. Endothelial cell proliferation is controlled by VEGFR-2. Beside others, VEGFR-2 is the most important signal transducer in angiogenesis and additionally, VEGFR-2 plays a major role in proliferation, migration, permeability, invasion and tube formation of endothelial cells [9, 45–48]. Thus, VEGFR-2 provides a possible target using antiangiogenic drugs in the treatment of gastric cancer.

Sunitinib is an orally available, TKI of VEGFR-1, − 2, and − 3, platelet-derived growth factor receptors (PDGF-R) α and β, KIT and other TKIs [49, 50]. In two phase II studies, using sunitinib in patients with advanced gastric cancer no benefit in survival could be observed. Besides that, patients in both studies suffered from severe adverse effects [51, 52]. Another randomized phase II trial combined sunitinib with chemotherapy in pretreated patients with unresectable or metastatic gastric cancer. Likewise, in the two studies using sunitinib as monotherapy for advanced gastric cancer, no significant benefit in survival or time to progression could be observed [53].

## Antiangiogenic agents in gastric cancer

Bevacizumab is a humanized monoclonal antibody blocking the binding of VEGF to its receptors. AVAGAST, a global, randomized, phase III trial, investigated chemotherapy with capecitabine xeloda/cisplatin versus chemotherapy with capecitabine xeloda/cisplatin plus bevacizumab. Beside PFS and overall response rate (ORR) showed significant improvements, the primary endpoint median OS was not reached (HR = 0.87; *p* = 0.1002) [54]. The AVATAR study, a Chinese, randomized, phase III trial, using the same study design, found no significant improvement in survival as well [55].

Ramucirumab is a humanized immunoglobulin G1 monoclonal antibody, showing promising antitumor effects in a number of malignancies [56]. The REGARD trial, a global, randomized, double-blind, phase III trial of 355 patients with progressive disease, investigated the addition of ramucirumab to standard chemotherapy. This addition brought a significantly prolonged median OS (3.8 to 5.2 month, *p* = 0.0473) [57].

The RAINBOW trial investigated ramucirumab as second-line treatment in patients with advanced gastric or gastro-esophageal junction cancer and disease progression after first-line chemotherapy, showing a significantly better OS in the ramucirumab plus chemotherapy group (median 9.6 vs. 7.4 month, *p* = 0.017) [58]. Due to these results, the FDA has approved ramucirumab in April 2014.

## Discussion

The role of targeted therapy in gastric cancer emerged over the last few years. HER2 status and angiogenesis, both associated with disease aggressiveness were recognized and established as prognostic markers. As shown and described in this study, preclinical as well as clinical trials have proved the importance of combining conventional chemotherapy and targeted therapy in gastric cancer.

Until now, trastuzumab remains the only monoclonal antibody showing significant benefits in gastric cancer. Due to the development of trastuzumab-resistance the positive effects are limited. Currently a number of promising molecules, showing synergistic effects in concomitant use with trastuzumab to overcome secondary resistance are under investigation. In the therapeutic field of angiogenic inhibitors, ramucirumab and apatinib, both VEGFR-2 inhibitors, remain the only two promising agents until now. As in HER2-directed targeted therapy, mechanisms for intrinsic or secondary resistance to anti-angiogenic therapy need to be unmasked.

Combined targeting of HER2 and VEGF showed encouraging inhibition rates in breast cancer. Due to these findings, further studies and trials, combining HER2 and VEGF-targeted therapies in gastric cancer are necessary.
